# Coronin-1C is a novel biomarker for hepatocellular carcinoma invasive progression identified by proteomics analysis and clinical validation

**DOI:** 10.1186/1756-9966-29-17

**Published:** 2010-02-24

**Authors:** Long Wu, Chun-Wei Peng, Jin-Xuan Hou, Yan-Hua Zhang, Chuang Chen, Liang-Dong Chen, Yan Li

**Affiliations:** 1 Department of Oncology, Zhongnan Hospital of Wuhan University and Hubei Key Laboratory of Tumor Biological Behaviors, No 169 Donghu Road, Wuchang District, 430071 Wuhan, PR China

## Abstract

**Background:**

To better search for potential markers for hepatocellular carcinoma (HCC) invasion and metastasis, proteomic approach was applied to identify potential metastasis biomarkers associated with HCC.

**Methods:**

Membrane proteins were extracted from MHCC97L and HCCLM9 cells, with a similar genetic background and remarkably different metastasis potential, and compared by SDS-PAGE and identified by ESI-MS/MS. The results were further validated by western blot analysis, immunohistochemistry (IHC) of tumor tissues from HCCLM9- and MHCC97L-nude mice, and clinical specimens.

**Results:**

Membrane proteins were extracted from MHCC97L and HCCLM9 cell and compared by SDS-PAGE analyses. A total of 14 differentially expressed proteins were identified by ESI-MS/MS. Coronin-1C, a promising candidate, was found to be overexpressed in HCCLM9 cells as compared with MHCC97L cells, and validated by western blot and IHC from both nude mice tumor tissues and clinical specimens. Coronin-1C level showed an abrupt upsurge when pulmonary metastasis occurred. Increasing coronin-1C expression was found in liver cancer tissues of HCCLM9-nude mice with spontaneous pulmonary metastasis. IHC study on human HCC specimens revealed that more patients in the higher coronin-1C group had overt larger tumor and more advanced stage.

**Conclusions:**

Coronin-1C could be a candidate biomarker to predict HCC invasive behavior.

## Background

Hepatocellular carcinoma (HCC), accounting for an estimated 600,000 deaths annually, is the third leading cause of cancer-related mortality worldwide [1]. Most cases occur in Asia and sub-Saharan Africa [2,3], however, the incidence is also expected to double over the next 10 to 20 years in the West, possibly due to the increased HCV infection [4]. While curative therapies are possible if the lesion remains early and localized, almost 70% of resected cases recurred within 5 years [5]. Although impressive progression has been made in providing an increasingly comprehensive portrayal of HCC [3,6,7], biomarkers that indicate the risk of invasion and metastatic potential of HCC and can be widely used in clinical settings are not currently available [8,9].

For a better insight into the characteristic of HCC metastasis, the stepwise metastatic human HCC cells MHCC97L and HCCLM9, with low and high metastatic potentials, were established via repeated in vivo selection and characterized by a similar genetic background but with significant differences in spontaneous metastasis behavior [10-12], providing appropriate model systems for comparative study on the molecular events correlated with HCC metastasis [13-15].

Plasma membrane, the structure surrounding all living cells and acting as the primary interface between the cellular contents and the extracellular environment, plays crucial roles in cell functions. Membrane proteins and other components maintain cell structure, motility and recognition [16] involved in receptor-binding and further transport of bound components into the cell, cell-cell and cell-matrix interactions, and the organization of the cytoskeleton [16-18]. The composition and characteristics of membrane proteins of tumor cells are modified during malignant transformation and make them likely candidates for cancer biomarkers [19]. Comparative proteomics with the recent advances are promising tools for discovering novel invasive and metastasis-associated candidate biomarkers of HCC.

The current work was to identify potential membrane proteins related to HCC invasive progression, using human HCC cells with different metastasis potentials, by proteomics analysis, experimental animal studies and clinical validation. To gain insights into potential candidate biomarkers contributing to invasion and metastasis, two well defined and unique HCC cells with multiple progressive and metastatic potentials, HCCLM9 cell with a highly lung metastasis rate 100%, and MHCC97L cell with a low lung metastasis rate 0% [12-14], were selected as our study models.

## Methods

### Cell lines and cell culture

The two cloned cell lines, MHCC97L and HCCLM9, are derived from the same host cell line MHCC97, in a process of cloning culture and 9 successive in vivo pulmonary metastases selection, as described previously [1,2]. These cells are cultured at 37°C in 5% CO2/95% air and RPMI 1640 (Sigma, USA) supplemented with 10% fetal bovine serum (Amresco, USA). Cells are grown to 80% confluence and passaged.

### Membrane proteins extraction

Membrane proteins from cultured cells were extracted using ProteoExtract^® ^subcellular proteome extraction kit (Cat. No. 539790, Merck, Germany) according to the protocol. All samples were stored at -80°C

### Sodium dodecyl sulfate polyacrylamide gel electrophoresis (SDS-PAGE)

After the BCA assay (Pierce, Rockford, IL) to quantify protein concentration, equal amounts of protein were loaded onto 12% gels (Invitrogen, Carlsbad, CA) and separated by SDS-PAGE. The gels were soaked in Coomassie brilliant blue dye overnight and excess stain was then eluted with a solvent (destaining).

### In-gel proteolytic digestion

The differential proteins band were excised manually from Coomassie brilliant blue stained gel with a disposable pipette, cut into small pieces, and transferred into 0.5 ml Eppendorf tubes. The gel pieces were destained by adding 60 μl acetonitrile/200 mM NH4HCO3 (1:1), vortexed 5 min, and centrifuged at 12,000 × g for 5 min and then the supernatant removed. This step was repeated until the gel pieces were completely destained. 60 μl acetonitrile were added, vortexed for 5 min, and centrifuged at 12,000 × g for 5 min and then the supernatant removed, this was repeated twice until the gel pieces were completely white. The gel pieces were dried, rehydrated, and incubated in 18 μl ice-cold trypsin solution (12.5 ng/mL in 0.1 M NH_4_HCO_3_) at 4°C for 20 min. The supernatant was removed and pipetted in 15 μl of the previous buffer without trypsin to maintain proteolytic digestion for 12 h at 37°C in a wet environment. We added 60 μl extract solution (5% formic acid in 50% acetonitrile) then sonicated the extract solution for 5 min. Peptides were collected in supernatant.

### Protein identification by ESI-MS/MS

ESI-MS/MS was conducted on a capillary system equipped with the Aksigent autosapmler(NanoLC-2D system, US.). A reverse-phase column (C18, OD = 360 μm, ID = 4.6 μm) was used to separate. The compartment of the autosampler was set at 10°C throughout the analysis. The mobile phase consisted of two components, with component (A) being 0.1% acetic acid and component (B) being 60% acetonitrile and 0.1% acetic acid. The solvent gradient was started from 5% B and held for 5 min, then programmed to 60% B in 40 min, and held for another 5 min, all at a flow rate of 300 L/min. MS-MS analysis were conducted on a Q-tof tandem mass spectrometer (Applied Biosystems, CA, USA). Positive ion mode ESI-MS was used for the analysis, with the TurboIonspray parameters optimized as follows: ionspray voltage (IS) 2200 V, declustering potential 60 V. The mass range chosen ranged from m/z 400 to m/z 1600. The ion source gas I (GSI), gas II (GSII), curtain gas (CUR), and the temperature of GSII were set at 40, 5, 30 and 175°C, respectively.

### Western blotting

After the BCA assay (Pierce, Rockford, IL) was used to quantify protein concentration, equal amounts of protein were loaded onto 12% gels (Invitrogen, Carlsbad, CA), separated by SDS-PAGE, and transferred to PVDF membranes (Immobilon 0.2 μm, Millipore, CA), which were then immersed in a blocking solution containing 5% skimmed milk and 0.1% Tween for 20 min. Afterwards, the membranes were washed and incubated with rabbit anti-coronin-1C (1:2000; Protein Tech Group, CA) or goat anti-integrin alpha 3 (ITGA3) (1:2000; Santa Cruz Biotechnology, Santa Cruz, CA) overnight at 4°C and then with goat anti-rabbit and rabbit anti-goat secondary antibody (1:3000; Protein Tech Group, CA) for 2 h at room temperature. Enhanced chemiluminescence (ECL; Amersham Biosciences, Piscataway, NJ) was used to visualize the immunoreactive bands. All bands were scanned and analyzed by Syngene GeneGenius bioimaging systems (Synoptics Ltd, UK).

### Animals and nude mice model of spontaneous pulmonary metastasis

Male athymic BALB/c nu/nu mice, 4 wks old, were obtained from Experimental Animal Institute of Hubei Center for Disease Control and Prevention and maintained in specific pathogen-free (SPF) condition at the Animal Experiment Center of Wuhan University. The facilities and the protocol of this experiment were consistent with the regulations on animal use for biomedical experiments issued by the Ministry of Science and Technology of China, and approved by the Animal Care Committee of Wuhan University. Both MHCC97L- and HCCLM9- nude mice were produced as described previously [12]. All mice were sacrificed under deep anesthesia by peritoneal injection of 3% phentobarbital chloride in approximately 6 wks after surgery. Liver samples were collected and stored at -80°C refrigerator. The lungs were fixed in 10% neutral formalin solution and processed for conventional pathological study to validate the model.

### Immunohistochemical staining of coronin-1C

Immunohistochemistry (IHC) was performed on 4-5 μm thick paraffin sections. Sections were deparaffinized and rehydrated with graded ethanols. For immunostaining, VECTORSTAIN ABC kit (Vector Lab, CA) was used according to the manufacturer's instructions. Primary antibodies used were rabbit anti-coronin-1C (Protein Tech Group, CA).

### Tumor development of spontaneous pulmonary metastasis in nude mice model of HCC

Highly spontaneous metastatic nude mice model (HCCLM9 group) of human HCC was used to study the relationship between coronin-1C levels and tumor progressive and metastasis. Twenty-four nude mice (HCCLM9) were produced as described previously. The mice were randomly divided into three groups of eight mice in each group. At the end of the fourth, fifth and sixth wk, one group of was sacrificed. Liver cancer and lung samples were stored -80°C refrigerator.

### Clinical validation

HCC specimens from 115 patients including 96(83.5%) males and 19(16.5%) females with mean age (M ± SD) of 47.9 ± 12.4 years (range 18-78) were obtained from Fanpu Biotech, Inc. All tumors were fixed with formalin and embedded with paraffin. Ten high power field of each tissue section were selected randomly and observed double blind by two investigators. The staining score of each section were calculated by staining intensity and positive rate of cancer cells. Staining intensity: the score of no staining, weakly staining and strong staining is 0, 1 and 2 respectively. Positive rate of cancer cells: 0-20% was recorded as 0; 20-50% was recorded as 1; >50% was recorded as 2. The sum of scores was computed as the score of staining intensity added the score of the positive rate of cancer cells. Then it was graded according the sum of scores: 0 (-); 1-2 (+); 3-4 (++).

### Statistical analysis

All the experiment data were integrated into a comprehensive data set. Numerical data were recorded directly. Chi-square test and Fisher's exact test were used to compare the clinicopathologic parameters among patients with different level of coronin-1C expression. Statistical analysis was performed on SPSS software version 13.0 (SPSS Inc. Chicago, IL), and *P *< 0.05 was considered as statistically significant.

## Results

### Differential expression of coronin-1C between HCCLM9 and MHCC97L cell strains as identified by ESI-MS/MS

**Figure 1:**
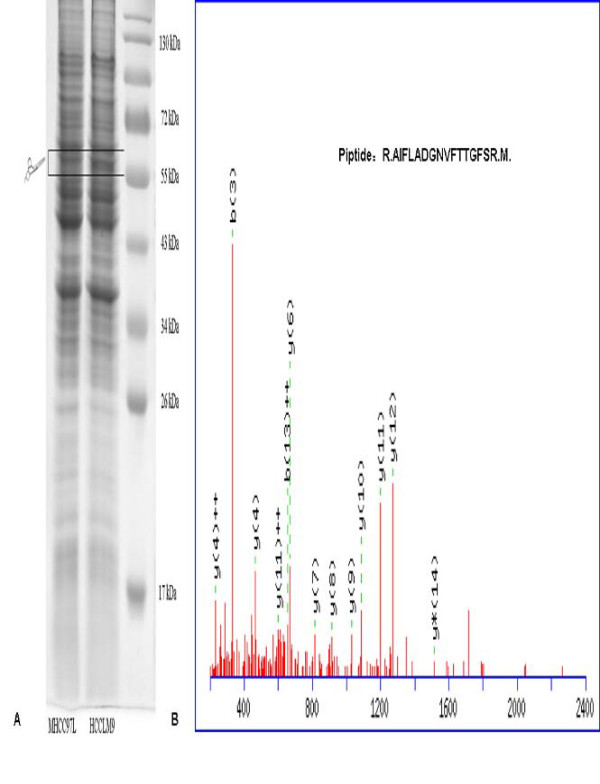
**Coronin-1C was identified as differentially expressed protein between HCCLM9 and MHCC97L cells**. **(A) **Membrane proteins were extracted from MHCC97L and HCCLM9 cells and compared by SDS-PAGE analyses. **(B) **The differential and interesting protein bands were excised and analyzed by ESI-MS/MS. One of MS/MS maps for Coronin-1C identification and the sequence of precursor were analyzed by MS/MS to be R.AIFLADGNVFTTGFSR.M.

Membrane proteins were extracted from MHCC97L and HCCLM9 HCC cells and compared by SDS-PAGE analyses [Fig. 1A]. The differential and interesting protein bands were excised and analyzed by ESI-MS/MS. A total of 14 proteins were identified by ESI-MS/MS among the differential bands [Table 1, Fig. 1A]. Coronin-1C, a promising candidate, was identified with high confidence [Fig. 1B].

**Table 1:** Differentially expressed proteins between HCCLM9- and MHCC97L -cell identified by ESI-MS/MS

Protein Name	Swiss-Prot Accession	Summary Score ^**a**^
Protein fto	Q9C0B1	84
UTP--glucose-1-phosphate uridylyltransferase	Q16851	78
Importin subunit alpha-1	P52294	71
1-acylglycerophosphocholine O-acyltransferase 1	Q8NF37	63
Tryptophanyl-tRNA synthetase, cytoplasmic	P23381	60
Proto-oncogene tyrosine-protein kinase Fyn	P06241	56
ERO1-like protein alpha	Q96HE7	55
EH domain-containing protein 1	Q9H4M9	54
RuvB-like 2	Q9Y230	53
Glycylpeptide N-tetradecanoyltransferase 1	P30419	49
U4/U6 small nuclear ribonucleoprotein Prp31	Q8WWY3	46
Copine-1	Q99829	45
Adenylyl cyclase-associated protein 1	Q01518	44
Coronin-1C	Q9ULV4	44

a Individual ions scores > 35 indicate identity or extensive homology, *P *< 0.05.

### Verification of coronin-1C differential expression by western blot

**Figure 2:**
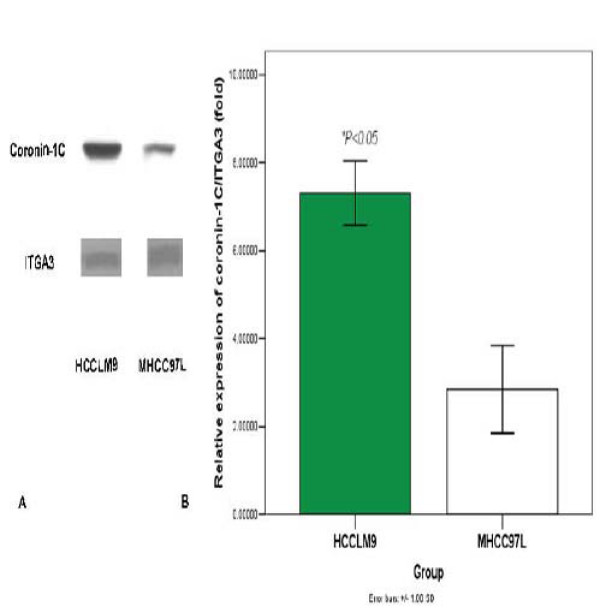
**Coronin-1C expression from membrane proteins of HCCLM9 cell rose significantly as compared with MHCC97L**. **(A) **Confirmation of coronin-1C expression by western blot analysis between HCCLM9 and MHCC97L cells. ITGA3, a typical membrane protein, was used as a control. **(B) **Densiometric scan of immunoblots shown in A.

Western blotting was conduced to further validate coronin-1C, as it has the advantage of enhanced sensitivity and specificity. ITGA3, a typical membrane protein, was used as a control. As our data show that coronin-1C from membrane proteins of HCCLM9 cells rose significantly as compared with MHCC97L [Fig. 2].

### Immunohistochemical staining (IHC) of coronin-1C in HCCLM9- and MHCC97L- nude mice model of HCC

**Figure 3:**
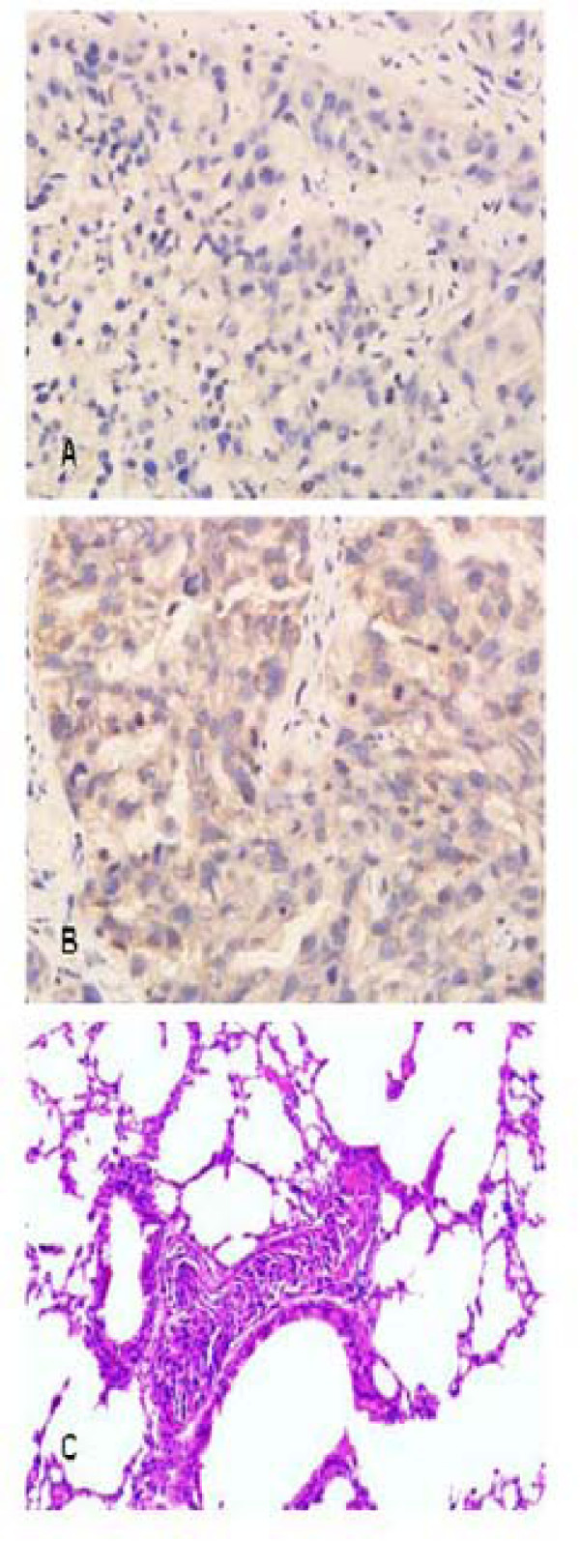
**Coronin-1C expression in HCCLM9- and MHCC97L- nude mice model of HCC**. Elevated coronin-1C expression was observed in liver cancer tissues of HCCLM9-nude mice. **(A) **Coronin-1C expression in tumor tissues of MHCC97L nude mice model of HCC by IHC. ×400; **(B) **Coronin-1C expression in tumor tissues of HCCLM9 nude mice model of HCC by IHC. ×400; **(C) **Spontaneous lung metastases occurred in HCCLM9- nude mice.

We had explored the relationship between coronin-1C expression and tumor spontaneous pulmonary metastasis in the nude mice model of HCC by IHC. Elevated coronin-1C expression was observed in liver cancer tissues of HCCLM9-nude mice [Fig. 3A, 3B], with highly lung metastasis rate 100% [Fig. 3C], compared with MHCC97L-nude mice, with no lung metastasis.

### Tumor development of spontaneous pulmonary metastasis in nude mice model of human HCC and tissues cronin-1C level

**Figure 4:**
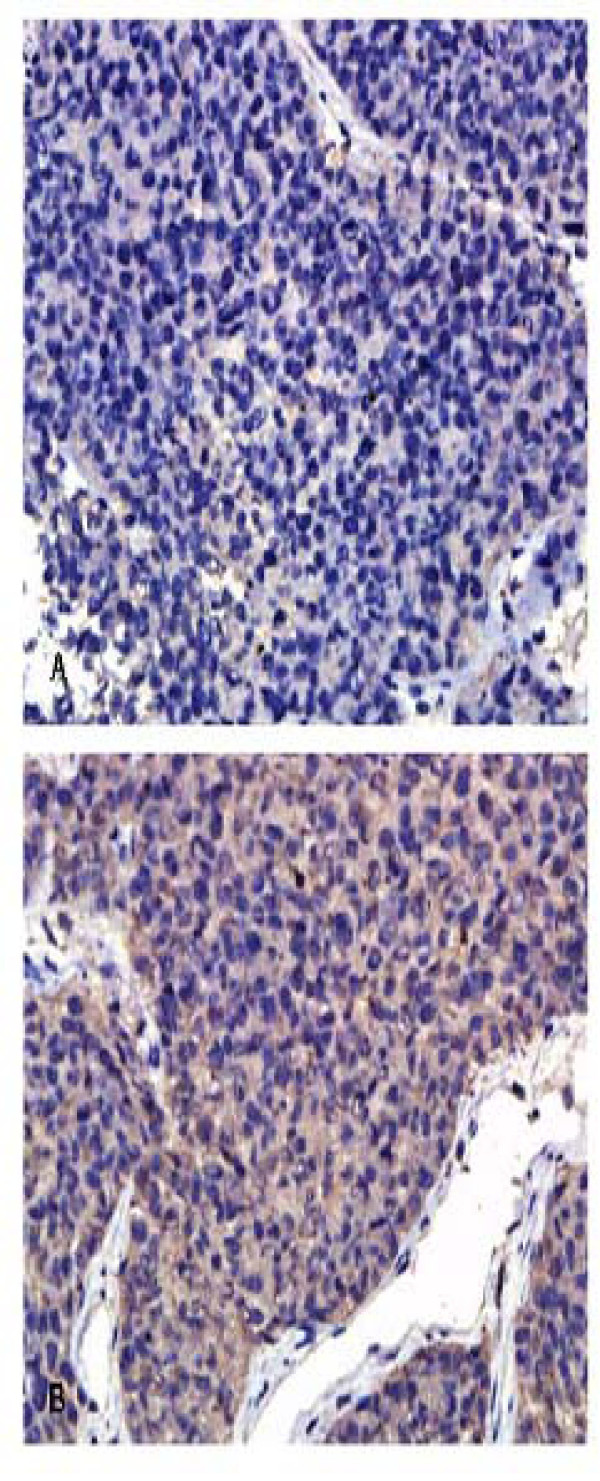
**Tissues coronin-1C level and development of spontaneous pulmonary metastasis in nude mice model of HCC**. Tumor tissues coronin-1C level rose remarkably at the end of the fifth wk. **(A) **Coronin-1C expression at the end of the fourth wk by IHC, ×400; **(B) **Coronin-1C expression at the end of the fifth wk by IHC, ×400;

We had investigated the relationship between cronin-1C expression and tumor spontaneous pulmonary metastasis in nude mice model of HCC. Tumor growth became accelerated from the third week on. No nude mouse had spontaneous pulmonary metastasis at the end of the fourth wk. On the fifth wk the mice showed signs of distress and histopathological study of the lungs showed conspicuous metastases. Simultaneously tumor tissues coronin-1C level rose remarkably, and representative images are presented in Fig. 4.

### Coronin-1C expression in HCC specimens

**Figure 5:**
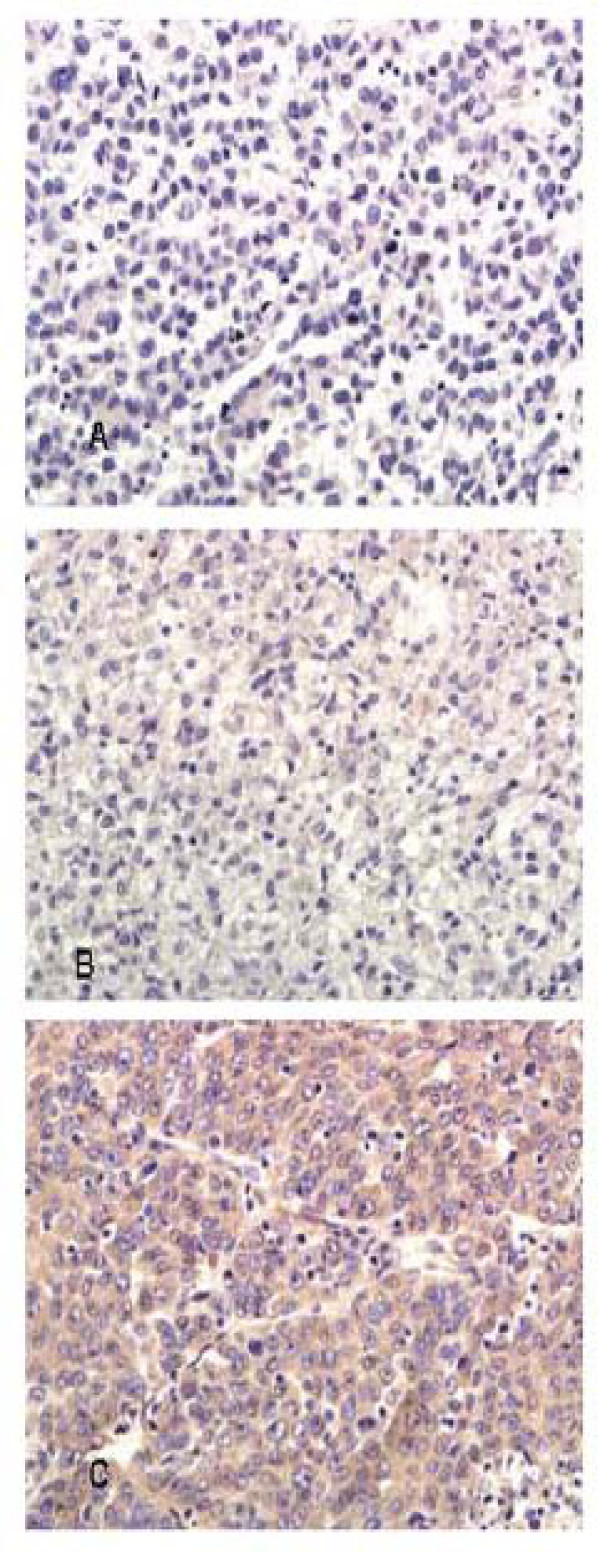
**The expression of coronin-1C human HCC specimens**. Significant differences in coronin-1C expression were observed among HCC specimens of different clinical stages. **(A) **Score-, ×400; **(B) **Score +, ×400; **(C) **Score ++, ×400.

We further investigated Coronin-1C expression in clinical HCC tissues using IHC analysis. Representative images are presented in Fig. 5. Coronin-1C was strongly stained (score ++) in 41 cases of the 115 samples (35.7%), weakly stained (score +) in 53 cases (46.1%) and not stained (score-) in 21 cases (18.3%). Significant differences in coronin-1C expression were observed among HCC specimens of different clinical stages. But there was no significant correlation between Coronin-1C expression with age and sex [Table 2].

**Table 2:** Correlation between tumor tissue coronin-1C expression and chinicopathological characteristics of 115 HCC patients

Clinicopathological characteristics	Coronin-1C expression n (%) ^**a**^			*P *value
		
	-	+	++	
**Age (years)**				
**> 50**	7 (14.6)	25 (52.1)	16 (33.3)	0.502 ^c^
**≤50**	14 (20.9)	28 (41.8)	25 (37.3)	
**Sex**				
**Male**	16 (16.7)	46 (47.9)	34 (35.4)	0.538 ^c^
**Female**	5 (26.3)	7 (36.8)	7 (36.8)	
**Tumor differentiation**				
**Well differentiation**	1 (8.3)	5 (41.7)	6 (50)	0.804 ^c^
**Intermediately differentiated**	16 (19)	39 (46.4)	29 (34.5)	
**Poorly differentiated**	4 (21.1)	9 (47.4)	6 (31.6)	
**Clinical Staging ^**b**^**				
**I+II**	17 (24.3)	33 (47.1)	20 (28.6)	0.047 ^c^
**III+IV**	4 (8.9)	20 (44.4)	21 (46.7)	

a The staining score of each section were calculated by staining intensity and positive rate of cancer cells.

b clinical staging are according to UICC cancer stage.

c Chi-square test and Fisher's exact test

## Discussion

Metastasis is a major cause of high mortality in HCC patients after surgical resection. To tackle the challenge, more prognostic biomarkers that could predict the progression and metastasis of cancer should be explored. Efforts have been made to find molecules of HCC progression and metastasis, including Alpha-fetoprotein (AFP) [20], Alpha-fetoprotein-L3 (AFPR-L3) [21], des-g-carboxy-prothrombin (DCP) [22], a-Fucosidase [23], Glypican-3 [24], transforming growth factor-b1 (TGFβ) [25], insulin-like growth factor-II (IGF-II) [26], insulin-like growth factor-binding protein-2 (IGFBP-2) [27], human cervical cancer oncogene (HCCR) [28], Golgi protein 73 (GP73) [29], hepatocyte growth factor (HGF) [30], KL-6 [31], vascular endothelial growth factor (VEGF) [32], and tumor-associated antigens (TAAs) [33]. However, HCC metastasis-associated indicators for clinical utility are still lacking. Advances have been made in genomics and proteomics to discover novel biomarkers for predication and diagnosis of cancer invasion and metastasis [34-37].

Our previous work applied two-dimensional gel electrophoresis (2-DE), matrix assisted laser desorption ionization/time of flight MS (MAIDLI-TOF-MS) and MS/MS to study the protemics profile differences between MHCC97L and MHCC97H [15]. Cytokeratin 19 was found to be correlated to HCC metastasis [15]. However, membrane proteins could be lost because of 2-DE innate limitations. The current study focused on membrane proteins, extracted from MHCC97L and HCCLM9 cells and compared by SDS-PAGE analyses. Among the differentially expressed candidate proteins, coronin-1C was found overexpressed in HCCLM9 cell as compared with MHCC97L cells, and further validated by western blot, animal model studies and clinical validations, suggesting that coronin-1C may be related to the metastasis phenotype of HCC.

Coronin is a major co-purifying protein identified from a cellular slime mold, Dictyostelium discoideum, localizing to crown-like structures on dorsal surface of a various cell types [18]. Coronins comprise at least seven members including coronin 1A, coronin 1B, coronin-1C, coronin 2A, Coronin 2B, and Coronin 7 [19]. Coronins play various roles in cell chemotaxis, cytokinesis, phagocytosis, locomotion and migration [38].

Located at cell pseudopodia and submembranous cytoskele, Coronin 1C is ubiquitously expressed and could be extracted from both the cytosol and the membrane fraction. As F-actin bundling and crosslinking protein [39], it is involved in F-actin-dependent processes at cell cortex. Absence of coronin-1C inhibits fibroblast migration as shown by Thal et al [40], who found significantly higher levels of coronin-1C expression in glioblastoma cells than low malignancy gliomas cells. Further, functional analyses by coronin-1C knockdown revealed the roles of coronin-1C in regulating cell proliferation, migration, invadopodia formation, and invasion in glioblastoma cells [40].

The current study found that coronin-1C expression in HCC nude mice models was correlated to the aggressive and metastastic behaviors of HCC. We further explored whether the detection of coronin-1C could help predict the development of spontaneous pulmonary metastasis in nude mice model of HCC. Coronin-1C level showed a marked upsurge at the end of fifth wk when pulmonary metastasis occurred, implying coronin-1C might indeed predict liver cancer progression and lung metastasis [Fig. 4].

Based on these findings, we focused on the relationship between coronin-1C and clinicopathological characteristics among HCC specimens. IHC study in 115 human HCC specimens demonstrated that patients with higher coronin-1C expression had more advanced stage, implying that increased coronin-1C could be involved in more aggressive growth of HCC.

In summary, the currrent work indicates the the role of coronin-1C in HCC aggressive and metastatic behavior. Coronin-1C level might reflect the pathological progression of HCC and could be candidate biomarker to predict HCC invasive behavior.

## Conclusions

Coronin-1C could be a candidate biomarker to predict HCC invasive behavior.

## List of Abbreviations

HCC: Hepatocellular carcinoma; SDS-PAGE: Sodium dodecyl sulfate polyacrylamide gel electrophoresis; SPF: Specific pathogen-free; IHC: Immunohistochemistry; ITGA3: integrin alpha 3; ECL: Enhanced chemiluminescence; AFP: Alpha-fetoprotein; AFPR-L3: Alpha-fetoprotein-L3; DCP: Des-g-carboxy-prothrombin; TGFβ: Transforming growth factor-b1; IGF-II: Insulin-like growth factor-II; IGFBP-2: Insulin-like growth factor-binding protein-2; HCCR: Human cervical cancer oncogene; GP73: Golgi protein 73; HGF: Hepatocyte growth factor; VEGF: Vascular endothelial growth factor; TAAs: Tumor-associated antigens; MAIDLI-TOF-MS: Matrix assisted laser desorption ionization/time of flight MS; 2-DE: Two-dimensional gel electrophoresis.

## Competing interests

The authors declare that they have no competing interests.

## Authors' contributions

LW carried out most parts of the experiment; C-WP, J-XH, Y-HZ, CC, and L-DC participated in the experiment; YL conceives the study project, organizes the whole study process, provides financial support, and finalizes the manuscript. All authors have read and approved the final manuscript.
